# Polygenic risk score opportunities for early detection and prevention strategies in endometrial cancer

**DOI:** 10.1038/s41416-020-0959-7

**Published:** 2020-07-06

**Authors:** Tracy A. O’Mara, Emma J. Crosbie

**Affiliations:** 1 Department of Genetics and Computational Biology, QIMR Berghofer Medical Research Institute, Brisbane, QLD Australia; 2 Division of Cancer Sciences, Faculty of Biology, Medicine and Health, University of Manchester, St Mary’s Hospital, Manchester, UK; 3 Division of Gynaecology, Manchester University NHS Foundation Trust, Manchester Academic Health Sciences Centre, Manchester, UK

**Keywords:** Risk factors, Endometrial cancer

## Abstract

Recent large-scale genetic studies, particularly genome-wide association studies (GWAS), have emphasised the importance of common genetic variation in endometrial cancer susceptibility. Although each of these variants only confer modest effects on endometrial cancer risk, together they are likely to explain a substantial amount of the familial relative risk of the disease. Therefore, methods to combine genetic risk variants, such as polygenic risk scores (PRS) have gained traction as an attractive method for individualised risk prediction and management. Here, we discuss the benefits of a PRS for endometrial cancer and considerations required for clinical implementation.

## Main

Endometrial cancer is the most commonly diagnosed gynaecological malignancy in the developed world.^1^ It is clear that endometrial cancer has a hereditary component, with studies estimating that women with a first-degree relative with endometrial cancer have a 2/3-fold increased risk of developing the disease themselves.^2^ A proportion of this can be explained by rare, pathogenic variants in high-risk genes, mainly the mismatch repair (MMR) genes associated with Lynch syndrome,^3^ while common genetic variants (i.e. polygenic factors) are estimated to account for around 28% of the familial risk of endometrial cancer.^4^

Significant progress has been made in our understanding of the role of common genetic variation in endometrial cancer susceptibility, with a recent review by Bafligil et al. highlighting genetic variants detected by genome-wide association study (GWAS) as showing greater reliability than those detected by candidate gene studies.^5^ GWAS studies for endometrial cancer have been largely driven by the international Endometrial Cancer Association Consortium (ECAC) and have identified 16 genetic regions associated with risk of this disease.^4,6^ Collectively, these regions explain approximately one quarter (~7%) of the polygenic risk of endometrial cancer.^6^

Individually, genetic variants identified by GWAS have a marginal effect on disease risk (odd ratios typically between 0.8 and 1.2). However, assessing genetic variants’ combined effect by polygenic risk score (PRS) has gained traction as a means to stratify patients into high- and low-risk strata. Theoretical PRS calculations by Bafligil et al., using 24 curated endometrial cancer genetic risk variants, predicted a 3.16-fold difference in endometrial cancer risk between women in the top 1% of the PRS distribution compared with the mean PRS.^5^

The results from Bafligil et al.^5^ are exciting and support further research into the development of a PRS for endometrial cancer. Studies to assess risk stratification of women in independent cohorts are currently underway. This work will refine the number of variants to include in PRS calculations for improved accuracy. Additionally, the integration of an endometrial cancer PRS with other known endometrial cancer risk factors (e.g. obesity) should further improve risk stratification accuracy.

The overall survival for endometrial cancer is similar to that observed for breast cancer (82% survival over 5 years).^7^ However, outcome is significantly worse for women with late stage disease (16–45% 5-year survival).^7^ Unlike many cancers, the incidence and mortality rates of endometrial cancer are increasing, probably due to the rising rates of obesity, sedentary lifestyles and the ageing population. These increases are projected to continue over the next decade, providing impetus for the development of early detection and disease prevention.^8^

An endometrial cancer PRS could potentially provide personalised risk information for a considerable proportion of women. Population-based risk management strategies could identify women at high risk of developing endometrial cancer for screening and allow for less monitoring for women with a low PRS.^9^ This would also provide opportunities for targeted interventions for high-risk women such as progestin (or progestin-based alternative) treatment and/or bariatric surgery, which have demonstrated remarkable success at endometrial cancer risk reduction.^10^ However, while the use of a PRS would provide access to personalised risk information to a wider group of women, careful consideration for implementation of the PRS into population-based screening will be required and large-scale studies necessary to assess its clinical impact.

The progress of PRS for endometrial cancer should be considered in the light of two major limitations. Firstly, GWAS for endometrial cancer to date have been unselected for subtype and consequently, the results from these studies are largely driven by the more common endometrioid endometrial cancer cases. Indeed, there are currently no genetic risk variants reliably associated with any non-endometrioid endometrial cancer subtype.^4,6^ This is an area requiring focus, given non-endometrioid subtypes of endometrial cancer are associated with poorer patient outcomes; these patients would greatly benefit from the early detection and preventative opportunities a PRS could provide. Secondly, all endometrial cancer genetic risk variants identified to date were discovered using European corhorts.^4,6^ Thus, PRS constructed using these variants may not provide suitable risk stratification in women of other ethnicities, reinforcing the need for future endometrial cancer GWAS to be performed in diverse population sets.

Despite these limitations, the potential benefits offered by a PRS to provide personalised risk assessment for endometrial cancer are exciting. While opportunities for population-based screening are evident, integration of a PRS could also be used in the familial cancer setting, to predict which women from Lynch Syndrome families are most likely to develop endometrial cancer and would benefit from prevention interventions. Larger GWAS meta-analyses planned by the ECAC will not only identify new genetic risk regions for endometrial cancer but also refine risk estimates associated with known regions, thus improving prediction accuracy of constructed PRS. It is likely that maximal benefit of an endometrial cancer PRS will be in combination with lifestyle and clinical measures.^9^ Assessment of the improved predictive value of these integrated risk models will be imperative in the development of risk stratification and screening programmes for endometrial cancer.^9^ “Is it possible to develop a personalised score that reflects a woman’s individual risk of developing endometrial cancer” emerged as the most important endometrial cancer research priority in a recently completed James Lind Alliance Priority Setting Partnership.^11^ Its endorsement by patients, the public and healthcare professionals supports the urgent need for a robust, clinically tractable endometrial cancer risk prediction model to improve outcomes from this disease.

## Data Availability

Not applicable.
